# CAR-T cell development for Cutaneous T cell Lymphoma: current limitations and potential treatment strategies

**DOI:** 10.3389/fimmu.2022.968395

**Published:** 2022-08-18

**Authors:** Van To, Vera J. Evtimov, Graham Jenkin, Aleta Pupovac, Alan O. Trounson, Richard L. Boyd

**Affiliations:** ^1^ Cartherics Pty Ltd, Notting Hill, VIC, Australia; ^2^ Department of Obstetrics and Gynaecology, Monash University, Clayton, VIC, Australia; ^3^ Australian Regenerative Medicine Institute, Monash University, Clayton, VIC, Australia; ^4^ The Ritchie Centre, Hudson Institute of Medical Research, Clayton, VIC, Australia

**Keywords:** CAR-T cells, CTCL, dual CARs, on-target/off-tumor toxicity, tumor heterogeneity

## Abstract

Chimeric antigen receptor (CAR)-T therapy has demonstrated remarkable outcomes for B cell malignancies, however, its application for T cell lymphoma, particularly cutaneous T cell lymphoma (CTCL), has been limited. Barriers to effective CAR-T cell therapy in treating CTCL include T cell aplasia in autologous transplants, CAR-T product contamination with leukemic T cells, CAR-T fratricide (when the target antigen is present on normal T cells), and tumor heterogeneity. To address these critical challenges, innovative CAR engineering by targeting multiple antigens to strike a balance between efficacy and safety of the therapy is necessary. In this review, we discuss the current obstacles to CAR-T cell therapy and highlight potential targets in treating CTCL. Looking forward, we propose strategies to develop more powerful dual CARs that are advancing towards the clinic in CTCL therapy.

## Introduction

Cutaneous T cell lymphoma (CTCL) is a heterogeneous group of T cell-derived non-Hodgkin lymphomas that affects the skin. Due to a lack of standardized treatments for all variants of this group of diseases and the indolent nature of early stages, CTCL carries a poor prognosis. If diagnosed early, CTCL can be managed by conventional treatments such as skin-directed therapies (topical steroids, chlormethine gel, phototherapy and local radiotherapy) or systemic therapies (interferon, extracorporeal photopheresis, retinoids, methotrexate, total skin radiotherapy, chemotherapy and allogeneic hematopoietic stem cell transplantation), but they often have short-lived success in patients with advanced-stage disease and, in turn, infections may contribute to the worsening of disease (1). Therefore, CTCL treatment remains challenging; effective therapies that elicit durable clinical responses remain a major unmet need.

A key hallmark of CTCL is severe progressive T cell immunodeficiency, which contributes to increased risk of infections (2, 3) that are the most prevalent cause of death in patients with advanced disease (3). Given the impaired patient immunity, increasing the anti-tumor response by immunotherapy is a fundamental and logical approach to combating this cancer and has revolutionized the treatment landscape for many CTCL patients. Monoclonal antibody (mAb) treatment has been the most promising therapy, as indicated by its approval from the Food and Drug Administration for the treatment of advanced-stage CTCL (4–7). However, toxicities associated with mAb/drug conjugation, limited tumor penetration, and high relapse rates occur in most treated patients (8), raising major hurdles to mAb targeted drug therapy. The logical extension to this approach is chimeric antigen receptor (CAR)-T cell therapy, which combines the tumor targeting of mAbs with the power of cell-mediated cytotoxicity. CAR-T cell therapy has now emerged as an effective adoptive immunotherapy, offering remarkable success in the treatment of blood cancers, particularly B cell malignancies (9). By design, CAR-T cells specifically target cancer *via* an extracellular antigen-binding domain. T cell receptor (TCR) activation and co-stimulation lead to the killing of the targeted cancer cell.

Despite their promise, developing CAR-T cell therapies for treating T cell malignancies, including CTCL, is challenging because of intra-tumor heterogeneity and a lack of tumor-specific targets that are less likely to have off-tumor effects. Tumor antigen escape constitutes another roadblock to targeted therapy, predisposing patients to therapy failure and cancer recurrence after a therapeutic response. In this regard, CTCL patients were observed to have downregulated expression of CD30 after brentuximab treatment; a similar phenomenon also occurred with C-C chemokine receptor type 4 (CCR4) following mogamulizumab treatment (10, 11). Similarly, CD19 negative malignancies are emerging following CD19 directed CAR-T cell therapy (9, 12). One potential strategy to overcome antigen escape, including in CTCL treatment, is to develop dual-specific CARs targeting two tumor antigens simultaneously. By engineering combinatorial targets, this therapeutic modality could:

1) circumvent cancer escape through mutational loss of a nominal target antigen,
2) capture different neoplastic subclones, and
3) maximize synergistic efficacy upon antigen engagement.

However, this must be balanced with the potential risk of increased on-target/off-tumor toxicity such as T cell aplasia. Therefore, the target selection and optimization of antigen recognition play a fundamental role in therapeutic efficacy. This review will discuss the obstacles of translating CAR-T technologies into an effective CTCL treatment and will propose strategies, focusing on multi-targeted CAR configurations, to successfully advance such a CAR-T cell therapy to clinical application.

## CTCL – The disease and the underlying tumorigenicity

Mycosis fungoides (MF) and Sezary syndrome (SS) are the most common subtypes of CTCL and constitute approximately 60% of the disease presentations. MF is an indolent CTCL subtype with different skin manifestations observed depending on the stage of the disease (13). It may initially present as cutaneous patches (reddish, purplish, or brownish in color and itchy), then develop into thicker, scaly plaques, finally turning into tumors at late stages. In contrast, SS is an aggressive subtype characterized by erythroderma with pruritus, lymphadenopathy, and atypical circulating lymphocytes considered as Sezary or Lutzner cells (13).

The origin of the malignant T cell transformation involved remains unclear. Whether malignant T cells in MF/SS travel from the primary tumor within the skin to the bloodstream, or whether tumors arise from disease cells in circulation and then traffic to the skin resulting in accelerated tumor outgrowth is at present unknown. It is suggested that MF is a chronic granulomatous response to persistent unidentified antigen, together with cytokine-driven immunological imbalances, which precede production of an atypical T cell clone (14). It is unclear how skin-homing lymphocytes are constitutively activated and transformed into malignant CD4^+^ T cells, but patients with CTCL cells are also observed to have an apparent loss of T cell repertoire complexity in peripheral blood (15). Moreover, a markedly decreased number of normal T cells occurs in CTCL patients, particularly those with malignant blood involvement (15). These findings indicate that the disease disrupts the entire T cell population and that the neoplastic population may also arise systemically rather than simply from a clonal expansion from skin only (16).

In spite of this, the disease immunophenotype (CD4^+^/CD7^-^/CD26^-^), has been widely used to evaluate circulating neoplastic T cells in patients with blood involvement (13, 17–19). Physiologically, CD7^+^ cells are induced into cell apoptosis *via* CD7 - galectin-1 interaction; therefore, CD7^-^ circulating tumors may acquire the ability to resist galectin-1-induced cell death in SS patients (20). CD26 cleaves and downregulates C-X-C motif chemokine 12 - a chemokine protein providing stimulatory signals for cell trafficking (21). Loss of CD26 on the circulating leukemic cells in CTCL increases C-X-C motif chemokine 12-dependent chemotaxis which, in turn, could promote malignant cell trafficking to the skin (21).

The nature of the signals emanating from the skin, which could induce extravasation of the leukemic-form of CTCL cells remains unclear. However, the immune imbalance caused by persistent exposure to foreign antigens, overexpression of skin-homing molecules, and misdirected interaction between innate and adaptive immune systems in cutaneous immune surveillance could result in severe inflammatory skin disorders, including malignancy (16). In CTCL patients, CD4^+^ helper T cells recovered from non-inflamed skin also display upregulated expression of the skin-homing molecules, cutaneous lymphocyte-associated antigen and CCR4 (22). This upregulated expression assists circulating CD4^+^ helper T cells to infiltrate and accumulate in CTCL skin lesions through the binding of their ligands (C-C motif chemokine ligand 17 and E-selectin) expressed on endothelial cells in cutaneous microvessels (16). Uninflamed skin in CTCL patients also contains allergen-specific T cells in the epidermis and dermis, which are often increased in inflammatory skin conditions such as atopic eczema (23). This suggests that skin-homing memory T cells can not only migrate to the dermis and epidermis in response to inflammation but may also home to the skin constitutively (23). The imbalance of skin defense responses may be critical to understanding how malignant skin-homing T cells arise. In the CTCL tumor microenvironment, T cell lymphoma cells are surrounded by abundant immature resident dendritic cells that are incapable of processing any suspected pathogens (24). Despite this, CD4^+^ T cells appear to be repetitively stimulated, facilitating pathological immune reactions such as autoimmune disease or even malignancy transformation (24).

## CAR-T therapy

**Figure 1:**
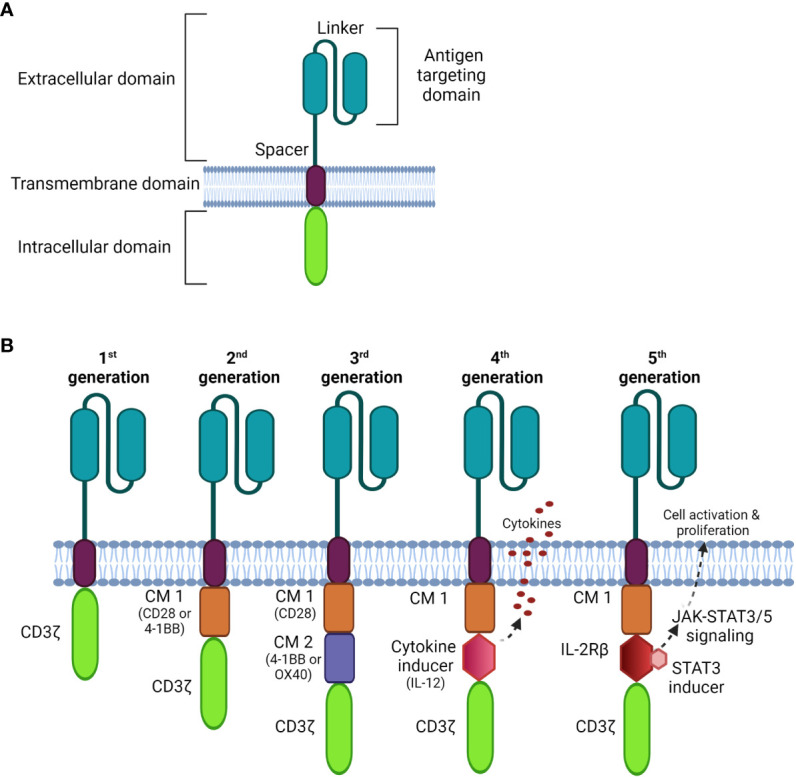
Schematic diagrams of CAR structures. **(A)** The basic structure of a CAR comprises the extracellular domain, a transmembrane domain, and the intracellular domain. **(B)** Comparison of four generations of CAR constructs. The first generation CAR comprises only the T cell signaling domain CD3ζ without the costimulation domain compared to the second generation, which includes one costimulatory molecule (CM); the third generation is generated with multi CMs, and the fourth generation is constructed with an additional cytokine transgenic gene to induce cytokines upon antigen engagement. The fifth generation comprises additional intracellular domains of cytokine receptors (e.g. IL-2Rβ) that allows JAK/STAT pathway activation to drive T cell activation and proliferation.

CARs are composed of four parts (**Figure 1A**): an extracellular antigen binding moiety, an extracellular hinge region, a transmembrane domain, and an intracellular T cell activation component (25). The antigen recognition domain, often consists of a single-chain variable fragment (scFv) that extends into the extracellular space allowing binding to antigen targets on tumor cells.

Recently, nanobodies have also been exploited as antigen-binding domains of CARs. These are composed of only two heavy chains, in which the target recognition module is composed of a single variable domain (VHH); they are smaller and more stable than conventional scFvs (26). Importantly, nanobodies can be easily humanized for therapeutic safety with comparable antigen-binding affinity and specificity to that of traditional scFvs (26–29).

The transmembrane domain anchors the CAR to the plasma membrane promoting the stability of antigen binding. It is usually derived from costimulatory molecules, such as CD28, to optimize intracellular signaling for CAR-T signal transduction. This complex is then connected to one or more signaling endodomains, which drive cell activation following the CAR-antigen interaction, triggering cytotoxicity to the target cancer cells.

Tumor cells undergo many mechanisms to evade immune attacks. One of these mechanisms is downregulation of the expression of major histocompatibility complex class I molecules, thereby reducing the overall antigen processing machinery (30). In this regard, CAR-T cells have advantages as therapeutics over conventional TCR-based immune reactions. Firstly, as modified TCRs, CARs have a superior antigen affinity to recognize and bind to unprocessed target antigens, which can potentially be of any chemical form (major histocompatibility complex-independent mechanism), whereas the TCR only recognizes peptide fragments presented in the context of major histocompatibility complex class I or II molecules. CAR-T cells thus have a greater capacity to identify the tumor *per se*, since most cancer antigens are not simply peptides. Secondly, CAR-T cells incorporate their own costimulatory domains, which augment anti-tumor immune responses to apoptosis and lyse target cells. Finally, the immune synapse forming at the interface between CAR-T cells and tumor cells enables greater toxicity than the respective antibody targeting (31).

There have been three previous generations of CARs shown in **Figure 1B**. More recently, fourth and fifth generation CARs (32, 33) were constructed with a transgenic cytokine or an intracellular domain of a cytokine receptor, such as an interleukin (IL)-2 receptor to:

1) increase selective anti-tumor cytotoxicity,
2) promote T cell activation and expansion,
3) recruit and activate innate immune cells to kill antigen-negative cancer cells, and
4) activate cytokine-driven signaling, which may increase the proliferative capacity and persistence of CAR-T cells.

Given the advantage in exhibiting more significant targeted-cytotoxic tumor killing than mAb therapy, CAR-T therapy has heralded a new landscape for the treatment of cancer, particularly in blood cancers. CD19-targeted CAR-T is a very successful therapy in eliminating treatment-refractory B cell lymphoma, demonstrated by impressive clinical responses (complete response rate: 70-90%) (34–37). As of May 2022, the Food and Drug Administration has approved six CAR-T cell products: three for B cell lymphoma and follicular lymphoma (axicabtagene ciloleucel, tisagenlecleucel, lisocabtagene maraleucel), two for multiple myeloma (idecabtagene vicleucel, ciltacabtagene autoleucel), and one for mantle cell lymphoma (brexucabtagene autoleucel) (9, 37–41).

## Hurdles and strategies in translating CAR-T therapy into the treatment of CTCL

While obtaining remarkable success in treating B cell malignancies, the application of the CAR-T approach to the treatment of T cell malignancies remains elusive. There are three main challenges to the use of CAR-T therapies for the treatment of CTCL. Firstly, given normal and malignant T cells have many phenotypic commonalities, if the target antigen is shared by healthy T cells, including those which form the CARs and tumor cells, the CAR-T cells would also kill them, resulting in T cell aplasia and immunodeficiency. Also, malignant T cells may be incorporated into the CAR-T cell product, which could facilitate their outgrowth and resist CAR-T cell treatment. Lastly, CTCL comprises different tumor subpopulations that enable the tumor to escape immune surveillance and mono-specific CAR-T cell killing.

### CAR targets expressed on both tumor and healthy tissue

**Figure 2:**
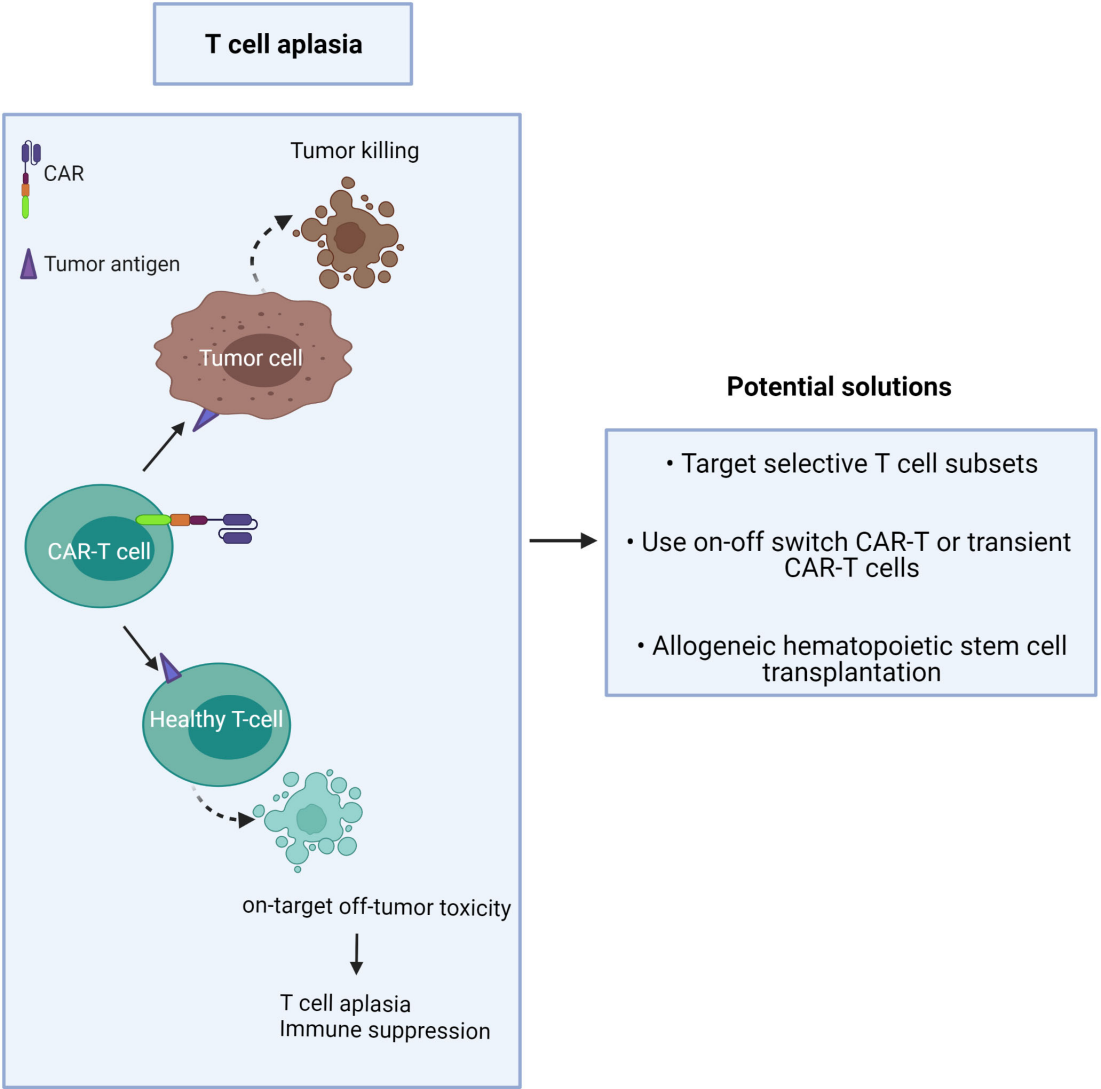
CAR-T cells induce on-target/off-tumor toxicity due to target antigen expression on healthy T cells.

Unlike B cell aplasia, which can be routinely treated by intravenous immunoglobulin therapy following CAR-T cell treatment (35), T cell aplasia is more challenging to manage as there is no available therapy presently to compensate for long-term T cell depletion (**Figure 2**). Patients with advanced-stage T cell malignancies often show T cell dysfunction, together with T cell aplasia after CAR-T injection, which may augment the risk of infection (3). It has been challenging to find unique CAR targets that are upregulated on only tumor cells but not expressed in normal tissue.

The proposed solution is to minimize toxicity and profound T cell deletion (**Figure 2**). This may be achieved by:

1) targeting CTCL tumor antigens co-expressed by minor T cell subsets or immunosuppressive T cells,
2) use of an ‘on-off switch’ in the CAR-T cell, or use of short-lived CAR-T cells,
3) providing T cell compensation by subsequent hematopoietic stem cell transplantation (42).

To limit inadvertent toxicities with long-lived CAR-T cells, transient CAR expression may be obtained through mRNA electroporation (43, 44) or through the incorporation of a safety switch (45–47). Preliminary data from clinical studies showed responses in patients and fully reversible toxicities (NCT03093168) (46). However, because of their short-term persistence, such CAR-T cells may not be sufficient to prevent tumor relapse and may require multi-dosing regimens to control the disease (44). To achieve a long-term cure by creating new patient-derived T cells, autologous hematopoietic stem cell transplantation is recommended following CAR-T cell treatment (48). The hematopoietic stem cells could be transduced to contain a CAR that would be expressed in the new T cells. Nonetheless, *de novo* production of T cells requires many months, if not a year or more, and is reliant on a functional thymus, which is often functionally depleted in adult patients (49). More recently, a suicide gene technology, known as the inducible caspase-9-based suicide gene (iCasp9), has been developed to eliminate CAR-T cells in case of severe toxicity. The iCas9 system consists of a drug binding domain linked to a human caspase 9 *via* a short linker (SGGGS) (50). When exposed to a dimerizer agent, iCasp9 is activated, triggering a signaling cascade that leads to DNA degradation and apoptosis of the transduced cells (51). Previous studies have shown that iCasp9 offers a better safety profile for CAR-T cells without detrimental impacts on the function of transduced cells (47, 52, 53). Transduction of T cells with a bicistronic vector encoding a CAR gene and suicide gene would avoid heterogeneous mixed products and ensures that transduced CAR-T cells express the safety transgene. However, a bicistronic cassette requires extensive design optimization of vector size, CAR scFv characteristics, and linker length to obtain a desired transduction efficiency and full antigen recognition. No “one size fits all” approach has thus been developed to date; therefore, integrated therapies should improve clinical benefit without cumulative toxicity when compared with any single-agent treatment.

The affinity of the binding domain also has a major impact on the efficiency and safety of CAR-T cells (54–57). For example, Chmielewski et al. showed that increasing the affinity of the CAR scFv did not improve the cytotoxicity against tumor cells but did increase the risk of on-target toxicity (57). In this study, CAR-T cells with high-affinity receptors decreased selectivity and killed normal cells, which express targeted antigens at physiological levels. In contrast, CAR-T cells with reduced affinity scFvs were activated exclusively by tumor cells which express high levels of targeted antigens. In line with this study, another group found that transducing peripheral blood lymphocytes with high-affinity TCRs increased on-target toxicity against healthy tissue with greater levels of cytokines (58). However, Lynn et al. found that high-affinity scFvs outperformed low-affinity scFvs in anti-tumor toxicity against leukemia xenograft mice (59). Also, other studies have raised the concern that weaker affinity CAR-T cells may affect T cell persistence and proliferation (60, 61). Therefore, tuning CAR affinity is crucial to achieving potent anti-tumor activity, while sparing normal tissue with lower expression of the targeted antigen. Given that normal and malignant T cells share many antigens, extensive optimization of CAR design is required to find the right balance between CAR-T efficacy and safety.

### Contamination of normal T cells with cancer T cells – a risk for an autologous CAR product

**Figure 3:**
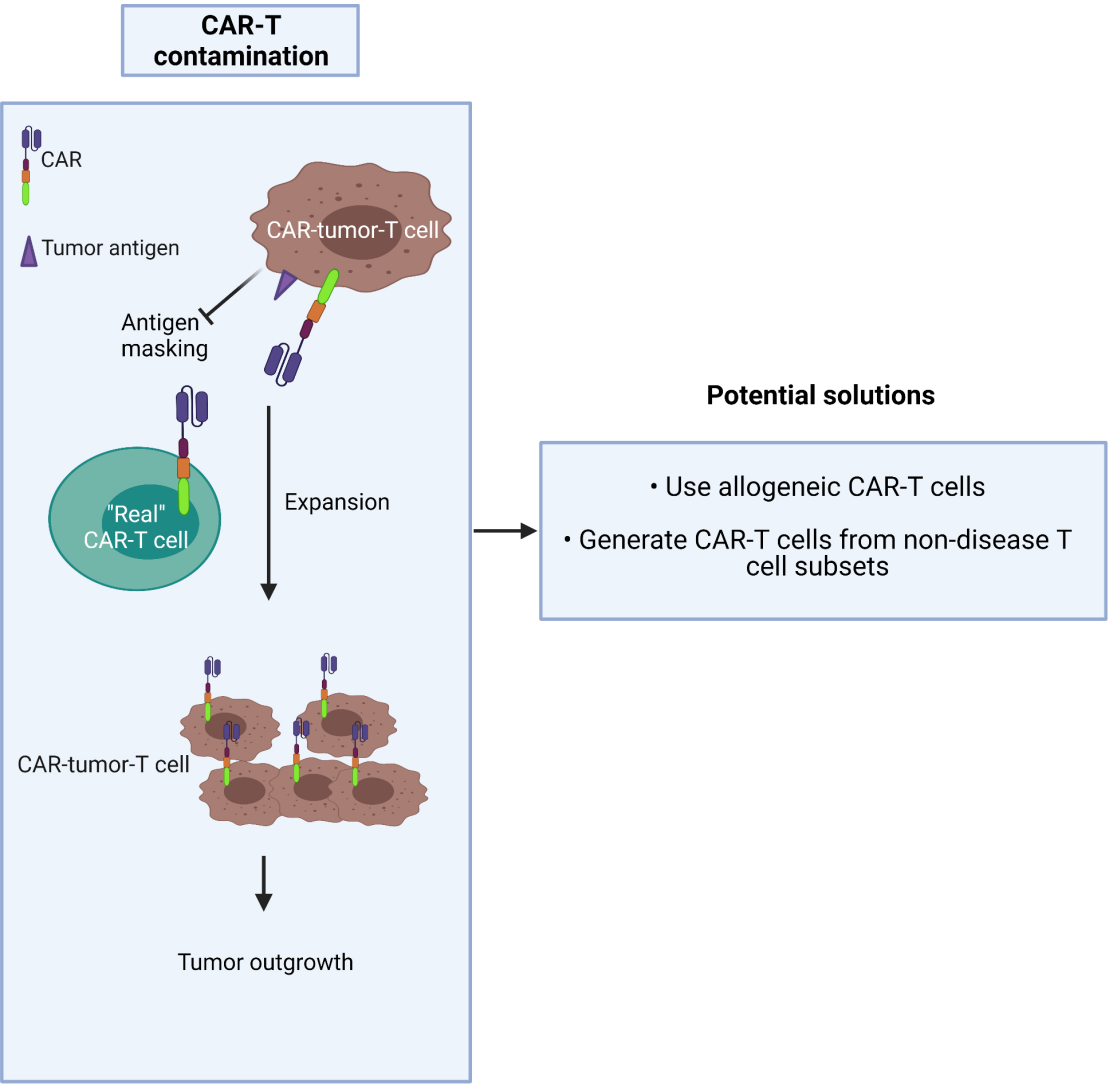
Malignant T cells may contaminate CAR-T cell products.

CAR-T production commonly involves a pan-T cell isolation step early in the process. Given that CTCL is derived from the CD4^+^ T cell subset, circulating tumor T cells may be collected during apheresis and transduced along with healthy T cells during CAR manufacture. The risk is that they may strongly proliferate after activation and exacerbate the outgrowth of the tumor in patients after being amplified in large numbers in *ex vivo* culture (62). The higher the tumor burden in blood involvement, the greater the risk of CAR product contamination, especially in advanced-stage patients who frequently have a high level of circulating malignant T cells. In this worst-case scenario, CAR-tumor-T cells may interact with tumor antigen on their own surface, resulting in a loop situation, which could mask the surface antigen from exposure to the “real” CAR-T cells (63) (**Figure 3**).

Two possible solutions to this dilemma are to either:

1) use allogeneic CAR-T cells (64) or
2) generate CAR-T cells from the non-disease T cell subset: i.e. purify and use the CD8^+^ population.

Disease cell contaminated products can be avoided by using allogeneic cells, however, this approach needs to be balanced with the risk of potentially life-threatening graft versus host disease whereby allogeneic T cells attack mismatched recipient’s tissues (65). This risk could be mitigated by matching patients to the allogeneic donor cells; however, it is not yet clear how long allogeneic CAR-T cells persist during remission in patients. Human leukocyte antigen (HLA) matching, expansion period and persistence are the subject of intensive study to develop a truly universal therapy.

In the second approach, a purification protocol is needed to prevent any malignant CD4^+^ T cells from being included in the final therapeutic cell product.

Previous studies have shown that tumor-stage MF patients, with a higher proportion of CD8^+^ tumor-infiltrating lymphocytes, including cytotoxic T cells, obtain a more favorable prognosis (66, 67). These data suggest that patients may benefit from CD8^+^ CAR-T infusion. One aspect to consider is whether CD8^+^ CAR-T cells alone are sufficient to kill tumor cells. Our group and others have demonstrated that CAR-T cells are capable of eliminating tumors whether CD4 or CD8 cells are used, however, activation-induced cell death may be more efficient by CD8^+^ CAR-T cells than CD4^+^ CAR-T cells (68–70). In this regard, CD4^+^ T cells have been shown to transmit helper signals to upregulate IL-15 expression, which in turn promotes the maintenance and function of memory CD8^+^ T cells (71). Also, memory CD8^+^ T cell generation depends on CD40–CD40 ligand interactions, which are triggered by CD4^+^ T cells (72). Memory CD8^+^ T cells have been shown to be more efficient in anti-tumor killing than naive cells by surviving longer, differentiating faster and secreting more cytokines (63, 72, 73). In another study, Sommermeyer et al. showed that combined CD4^+^ and CD8^+^ CAR-T cells exerted greater cytolytic activity for B cell lymphoma eradication *in vivo* compared to the CD8^+^ T cell subset alone, while the CD19 CD4^+^ CAR-T cells were incapable of eliminating the lymphoma in this tumor model (74). The enhanced efficacy in CD8^+^ CAR-T cells was attributed to IL-2 produced by CD4^+^ T cells, which may augment CD8^+^ CAR-T cell proliferation (74).

Variation in CAR-T efficacy also correlates with CD8^+^ T cell subtypes: CAR-T cells derived from CD8^+^ naive T cells and CD8^+^ central memory T cells secrete more cytokines and confer longer survival than those derived from CD8^+^ effector memory T cells, at least in an animal model (74). In CTCL patients, Horna et al. noted a prominent central memory population of CD8^+^ T cells in blood-involved CTCL patients (75), suggesting that CAR-T cells, generated from an autologous CD8^+^ T cell population, may offer a promising anti-tumor immune response and long-term persistence in patients.

However, an important consideration is whether these cells derived from the patients are sufficiently functional, since most preclinical studies generate CAR-T cells from healthy donor T cells which won’t have been exposed to chemotherapy in contrast to those collected from cancer patients. Hoffmann et al. showed the expansion of CAR-T cells significantly differed between two groups, where CAR-T cells from healthy donors expanded 6 times greater compared to those generated from patients (76). This implies that patient CAR-T cells may require a longer expansion period or optimized culture conditions to promote their proliferation. Future studies, focusing on the heterogeneity in the T cell composition and *ex vivo*-cultured conditions are thus required to maximize autologous CAR-T efficacy against CTCL.

### Fratricide of CAR-T cells

**Figure 4:**
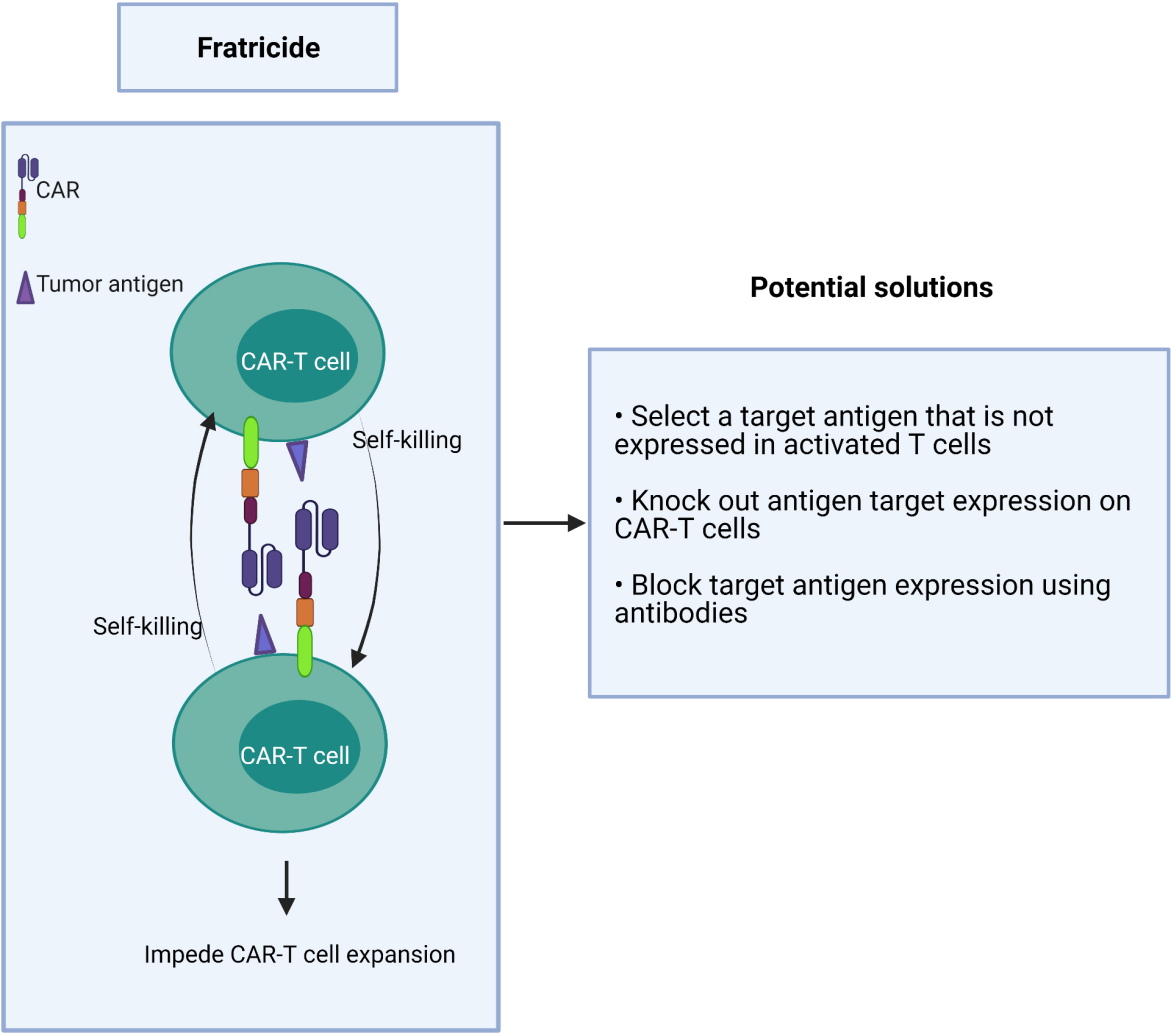
Fratricide between CAR-T cells greatly impedes expansion of the therapeutic product.

If a target antigen is shared between T effector cells and malignant T cells, it may cause self-killing of the CAR-T cells in a process known as fratricide (**Figure 4**). This “auto” killing greatly impedes the expansion and persistence of adoptive CAR-T cells during their manufacture (77–79).

Strategies to overcome this hurdle include:

1) choosing a target antigen that is not expressed or is downregulated in activated T cells during manufacturing,
2) genetic deletion of the target antigen on CAR-T cells *ex vivo*, and
3) the use of antibodies to block the target antigen during the CAR-T cell expansion to prevent binding of the CAR scFv which, in turn, prevents target-driven fratricide (**Figure 4**).

A disadvantage of this approach is that the final product requires extensive washing steps before harvesting. Otherwise, the residual antibody could compete with CAR-T cells in binding target tumors and reduce CAR-T cell efficacy. Previous studies, however, have successfully used antibody blockade to prevent T cell fratricide and to improve the cell yield during large-scale expansion (80, 81). Target-driven fratricide will also depend on the specific T cell subsets that the CAR-T cells are derived from; obviously avoiding using those expressing the target antigen is logical. In this regard, previous studies have shown that knocking out target antigen expression in CAR-T cells can render them fratricide-resistant. For example, upon CD7 gene disruption by CRISPR/Cas9, CD7^+^ CAR-T cells demonstrated complete fratricide resistance without compromising cytotoxic function against acute T cell leukemia (82–84). Similar approaches, which are well reviewed by Kozani and colleagues, were reported when CAR-T cells were engineered to target other pan-T antigens, such as CD3 and CD5 (85). One such approach used to overcome CD5 CAR-T cell fratricide is the use of a Tet-OFF expression system, which can be employed to control CAR expression in a reversible manner using doxycycline (86). In this system, the presence of doxycycline prevents a transactivator from binding the CAR promoter, thereby suppressing CD5 CAR expression during *ex vivo* expansion, but is restored following doxycycline withdrawal. This system enables greater CAR-T expansion and improves the survival of leukemia-bearing mice, however, more studies are required to validate the long-term persistence of CAR-T cells with engineered target antigens. Additionally, whether the Tet-OFF system induces an immune response in humans may be a crucial factor in determining how safe the therapy will be in clinical studies.

### Heterogeneity of the disease – the need for targeting multiple antigens

MF and SS have been characterized as two distinct T cell subtypes with disparate clinical behaviors (87). Furthermore, within individual SS patients, divergent subclones arise which increase with disease progression from early (IA and IB) to late (>IIB) stages (88). This high intra-patient variability indicates the evolution of phenotypic plasticity of Sezary cells during the disease. Oncogenomics and gene expression analysis reveal the underlying variability of T cell precursors between CTCL subtypes (89–91), which partly explain the difference in clinical manifestation in patients with MF and SS disease. Clonal malignant T cells from MF exhibit the phenotype of skin resident effector memory T cells (CCR4^+^ and cutaneous lymphocyte antigen^+^), while from SS they exhibit a phenotype of central memory T cells (CCR7^+^, CD27^+^, and L-selectin^+^). The latter have a greater capacity to proliferate and travel between the blood and lymphatic circulation (92). Crucially, patients were also observed to have different proportions of central memory and naive T cells in malignant T cells within the same SS disease, reflecting the heterogeneity of circulating malignant cells (93).

**Figure 5:**
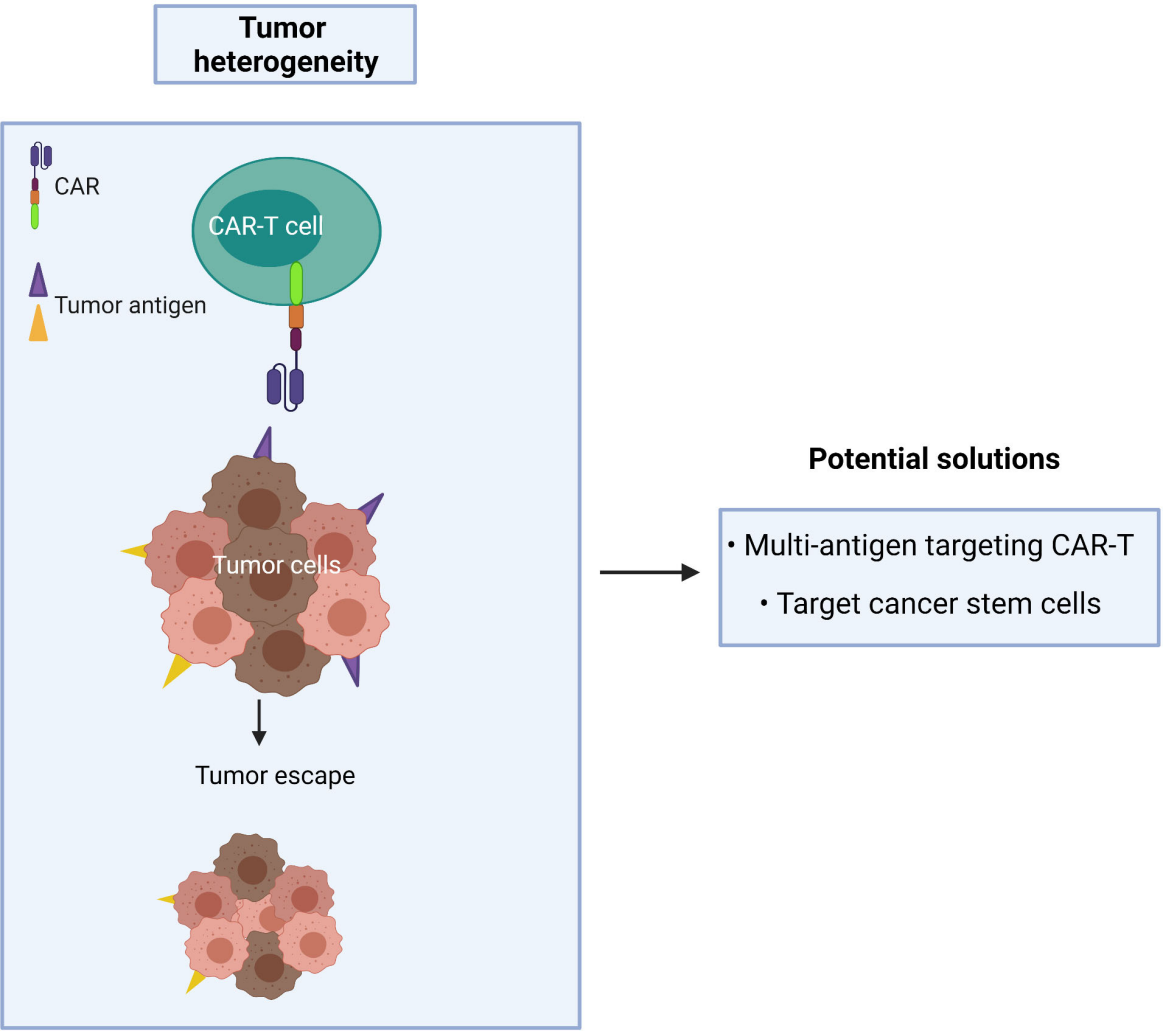
Cancer recurrence occurs when tumor heterogeneity resists single antigen targeting CAR-T treatment.

A CAR targeting a single tumor antigen may be insufficient to recognize the majority of tumor cells due to phenotypic heterogeneity as found in CTCL (**Figure 5**). An alternative approach, such as multi-antigen targeting, offers a promising strategy to mitigate antigen escape and hence obtain a broader spectrum of tumor clearance. Synergistic activation of the CAR-T effector cell can also be achieved when both CARs simultaneously engage with tumor antigens (94). However, while CAR-T cells may exhibit a greater ability to recognize tumor cells with multi-antigen recognition motifs, this may lead to an increased risk of on-target/off-tumor toxicity in patients. As T cells require three signals for full physiological activation (antigen binding, co-stimulation and cytokine signaling), the effector function of CAR-T cells relies on optimizing the components of each single CAR construct.

## Multi-targeted CAR-T cell strategy and potential targets

**Figure 6:**
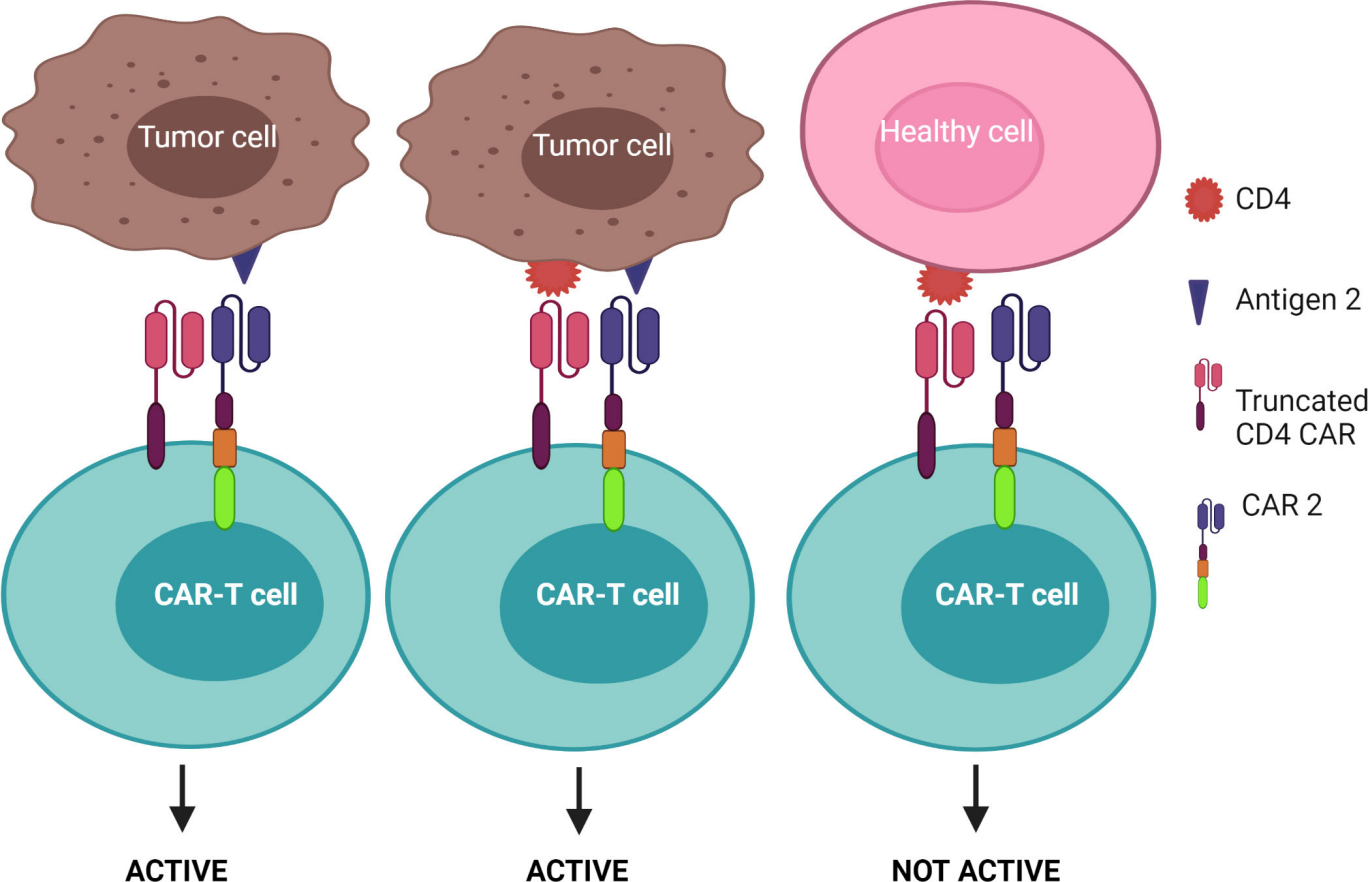
“HELP” logic-gated CAR-T cells for improving tumor recognition and alleviating on-target/off-tumor cytotoxicity on CD4^+^ healthy T cells. Truncated CD4 CAR directs CAR-T cells to CD4^+^ cells but does not trigger CAR-T cell activation without the presence of the second antigen.

**Figure 7:**
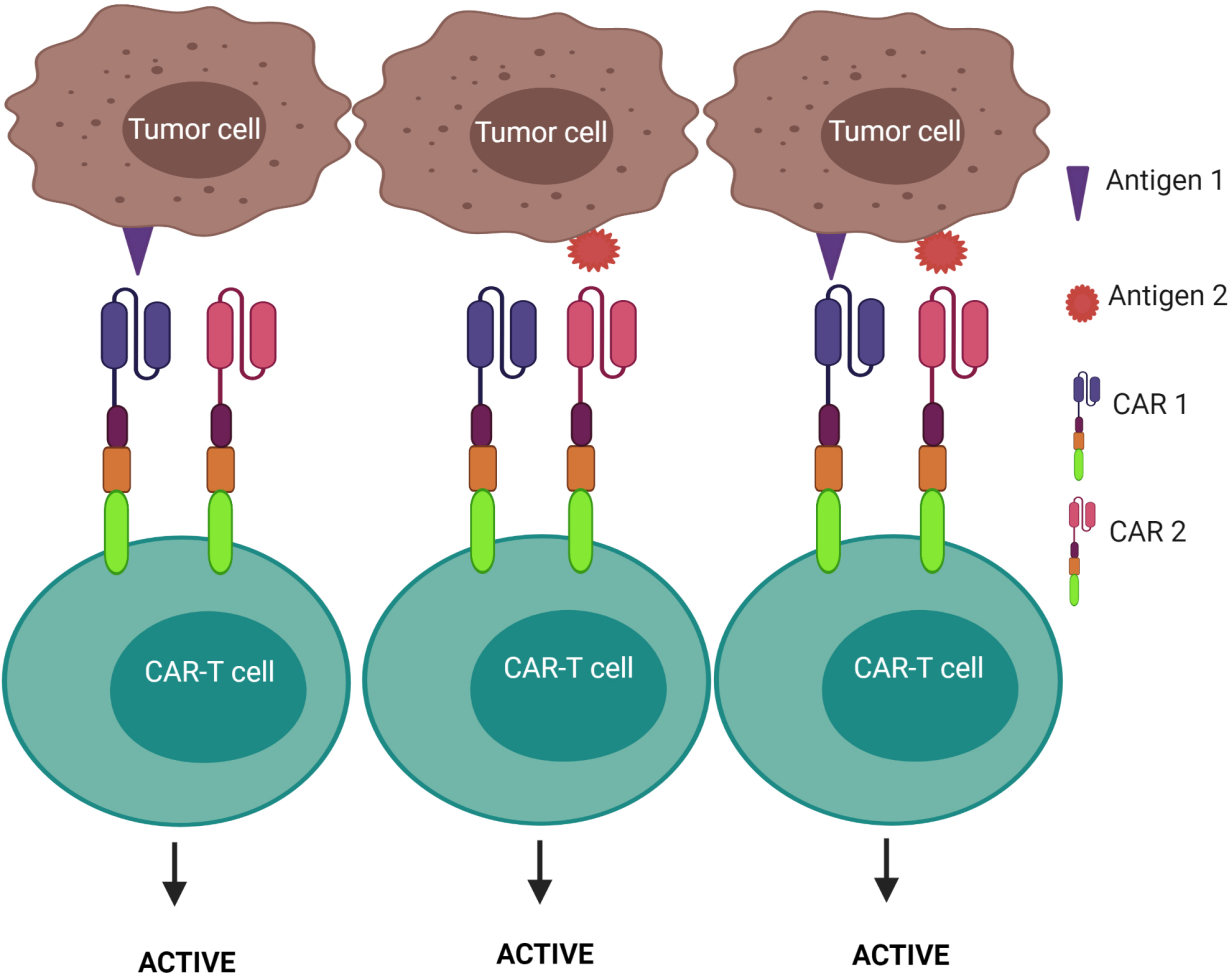
“OR” logic-gated CAR-T cells for improving the coverage of tumor population and preventing tumor escape. CAR-T cell activation is triggered when either antigen 1 or antigen 2 is encountered.

**Figure 8:**
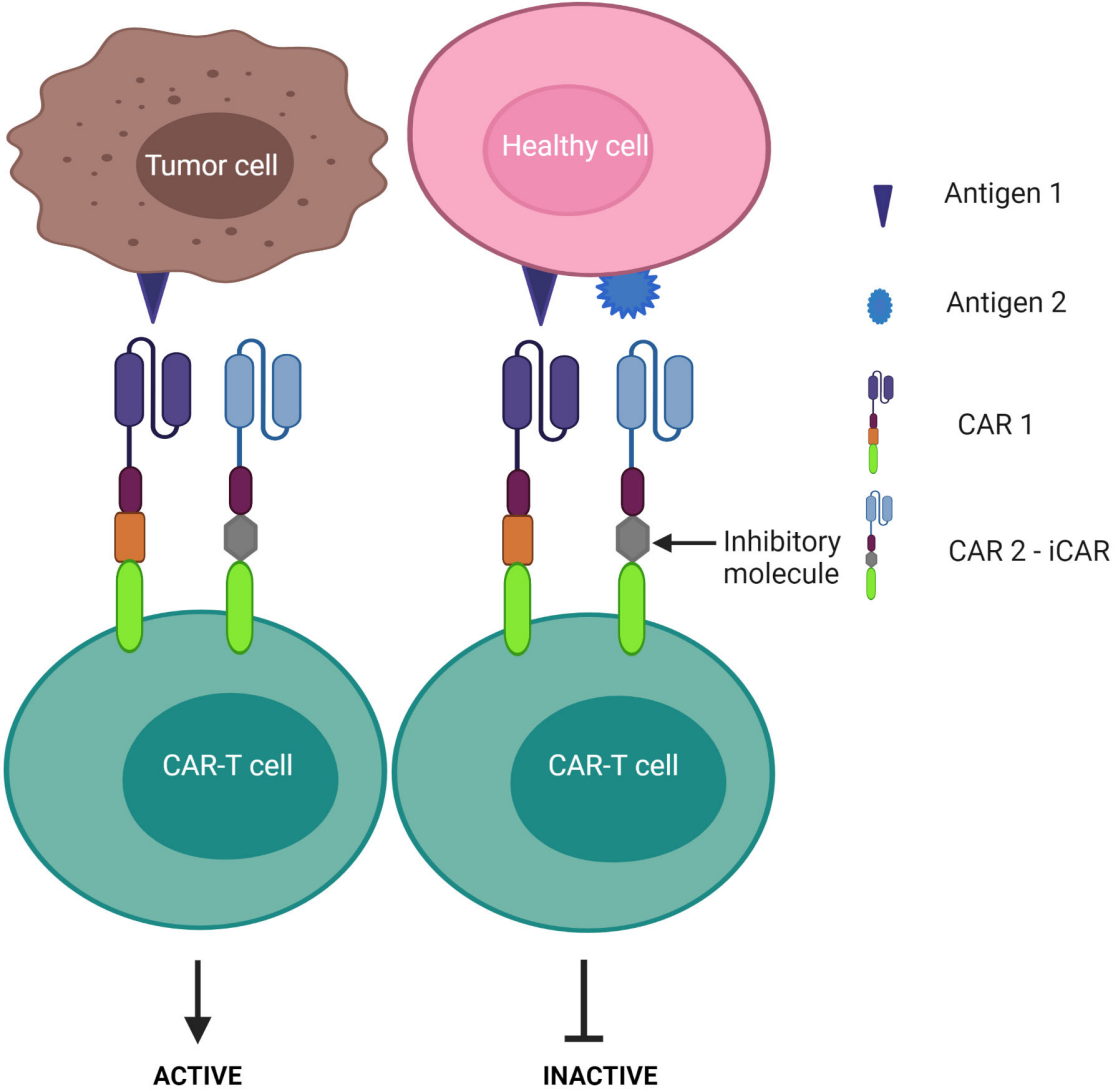
“NOT” logic-gated CAR-T cells selectively kill tumor cells expressing only tumor antigen 1 while the second CAR functions as an iCAR to inhibit CAR-T killing activity against antigen 1-positive normal cells.

Several main approaches are possible for establishing multi-targeted CAR-T cells, including the following:

1) CAR-T cells are only activated upon the engagement of the second CAR with antigen 2, while the first CAR is exploited to assist the binding of the second CAR with tumors – known as the “HELP” logic gate (**Figure 6**). The first targeted antigen, CD4, for example, is strongly expressed on both tumor cells and normal cells whereas the second antigen should be tumor specific. This approach uses the advantage of the first antigen’s overexpression on tumor cells to design a cognate CAR that assists CAR-T cell binding to tumor cells on which the second antigen is expressed. In this approach, CAR-T cells are only activated when the second antigen is engaged, as the first CAR (CD4 CAR) is truncated - devoid of a costimulatory activation motif, so it is incapable of activating the CAR-T cells.
2) CAR-T cells are activated after engagement with either antigen 1 or antigen 2 – known as the “OR” logic gate (**Figure 7**). Each CAR has its own signal domain, which is sufficient to trigger CAR-T activation, therefore, it would increase the chance of tumor recognition and prevent tumor escape.
3) CAR-T cells are inactivated if both antigens are engaged – known as the “NOT” logic gate (**Figure 8**). The inhibitory CAR (iCAR) enables CAR-T cells to distinguish between the tumor cells from the off-target cells.

Depending on antigen targets, a multipronged CAR-T construct can be optimized to strike a balance between efficacy and safety. Selecting the CAR target plays a fundamental role in the efficacy and safety of the therapy. Ideally, the target antigen should have:

1) high coverage of the tumor cell population(s) to achieve a broader spectrum of tumor clearance,
2) high specificity for tumor cells (particularly any cancer stem cells) to avoid on-target/off-tumor toxicity on normal tissue, and
3) high stability of expression to enable the CAR to achieve a long-term effect. The less stable the target antigen is, the greater chance of tumor escape.

It is often difficult to identify an appropriate target satisfying all the above-mentioned criteria. However, several antigens have been deployed as potential CAR targets and have gained initial encouraging outcomes in the preclinical and clinical treatment of CTCL. Depending on the density and specificity of target antigens, different antigen combinations can be exploited for more effective and safer CAR-T therapy. **Table 1** provides a summary of potential CAR targets and their potential logic gate inputs.

**Table 1:** Potential CAR targets and their respective logic gate strategies to enhance the recognition and precision of CAR-T therapy in CTCL treatment.

CAR targets	Logic gates
CD4	“HELP” logic gates
CD47	
CD30	“OR” logic gates
CCR4	
TAG-72	
CD37	“NOT” logic gate

## “HELP” logic gates

### CD4-targeted CARs

Despite its broad expression on functionally important healthy cells, the clonal expansion of CD4^+^ T cells in MF/SS makes CD4 a potential target for CARs (95). Phase 2 clinical studies have investigated the efficacy of a CD4 mAb (zanolimumab) for CTCL. High response rates were observed in patients with MF, whereas the result was suboptimal for SS patients (96). In a preclinical study, CD4 CAR-T cells killed the CD4-expressing leukemic cell line (KARPAS 299) and CD4^+^ malignant cells obtained from patients with SS (97). After four rounds of administration in mouse models, CD4 CAR-T cells suppressed the proliferation of T cell lymphomas and prolonged mice survival, compared to the control group (97). Phase I clinical trials (NCT03829540, NCT04219319, NCT04162340) have now been initiated to evaluate the safety and tolerability of anti-CD4 CAR-T cells in T cell malignancies, including T cell leukemia and T cell lymphoma; the outcomes have not yet been reported.

Since CD4^+^ T cells represent approximately two-thirds of normal T cells and are crucial for defending the body against infection (and cancer), CD4 CAR may cause non-specific toxicity on healthy CD4^+^ T cells and lead to severe immunodeficiency akin to human immunodeficiency virus/acquired immunodeficiency syndrome (98). Such CD4^+^ based immune deficiency often facilitates bacterial infection (3). Furthermore, although the aforementioned CD4 targeting mAb was well tolerated by CTCL patients (96), severe on-target adverse effects can occur using CARs as they are often more sensitive to lower levels of target antigen than the respective mAb. Thus, the incorporation of a safety switch, such as an elimination marker into CAR-T cells allows for their irreversible depletion during off-target or adverse events. For example, CAR-T cells can be engineered to co-express the truncated human epidermal growth factor receptor or CD20, which allows for selective elimination of the transgene-expressing CAR-T cells through the infusion of an associated mAb (cetuximab for epidermal growth factor receptor and rituximab for CD20) (99–102). These elimination markers, however, should not be expressed on normal tissue to avoid antibody-dependent cellular cytotoxicity. Additionally, approaches using transient CAR expression are also preferable to ensure that T cell immunity is restored and may offer a safer T cell therapy. This approach was tested by Ma et al. when they administered alemtuzumab, a mAb against CD52 - an antigen expressed by T cells, to delete CD4 CAR-T cells in treated mice (103). Within 6 hours of alemtuzumab administration at a safe human equivalent dose, over 95% of infused CD4 CAR-T cells were depleted from circulation in mice. (103). However, before implementing this approach in clinical studies, further research will need to elucidate the time point at which to administer alemtuzumab as this is crucial to give the CD4 CAR-T cells enough time to kill tumor cells before being neutralized, but also restrict toxicity caused by CAR-T therapy after tumor clearance. Further, designing an additional target to make up the “HELP” logic gate may mitigate “on-target” toxicity. As mentioned previously, CAR-T cells are only activated when the second antigen is engaged. However, once the CAR-T cell is activated through the signaling CAR, which may be mediated through enhanced tumor cell binding by engaging CD4, the CAR-T cell can induce the killing of adjacent tumor cells lacking CD4, but expressing the desired tumor-specific antigen. As indicated in **Figure 6**, this strategy may increase the safety profile of the CD4 CAR-T cell product as it facilitates enhanced binding of the CAR-T cells to CD4^+^ tumor cells, thereby improving the chance of additional target engagement and killing of the tumor cells.

### CD47-targeted CARs

CD47 is an integrin-associated protein that may be expressed on all nucleated cells and red blood cells (104, 105). Upon binding with the signal regulatory protein-α (SIRP-α), a protein strongly expressed on macrophages, CD47 transmits a “don’t eat me” signal to inhibit macrophage phagocytosis of normal cells (106). Many cancers have hijacked this system of cell survival by upregulating CD47 expression. In particular, hematopoietic cancers strongly express CD47 to evade immune destruction (106, 107). By inhibiting the “don’t-eat-me” signal, the anti-CD47 antibody causes phagocytosis of leukemia cells, eradicates T cell acute lymphoblastic leukemia engraftment, and leads to disease remission in a mouse model of leukemia (106). In addition, the anti-CD47 antibody synergizes with other antibody-targeted drugs to increase phagocytosis of non-Hodgkin lymphoma cell lines (Raji, SUDHL4, and NHL17 cells). In mouse lymphoma xenograft models, 89% of mice (8 out of 9 mice) showed more than 4 months of disease-free survival after treatment with an anti-CD47 antibody (108). In CTCL, CD47 is overexpressed in advanced MF and SS patients where it works in tandem with thrombospondin-1 to, not only allow for immune evasion, but also promote tumor cell migration and survival (107, 109). Blocking the CD47 receptor by soluble SIRP-α, (a thrombospondin-1 family member, CD47 decoy receptor) restored the macrophage phagocytosis of Sezary cells, resulting in tumor load reduction in peripheral blood (109).

Collectively, these data suggest CD47 is a promising CAR target in CTCL treatment. Despite CD47 CAR-T effectiveness in xenograft mouse models with solid tumors (110), no published studies have examined CAR-T cells targeting CD47 for the treatment of CTCL patients. However, given the broad expression of CD47 in normal tissues (104), the safety aspect of CD47 CAR-T cells therapy needs to be extensively tested. As with targeting CD4, designing multipronged CAR-T cells under a subsequent “HELP” gate framework, where a CD47 CAR assists T cells to bind with tumor cells first, followed by the ligation of the second CAR with another target antigen, may be possible. In this system, CAR-T cells are only capable of driving cytolytic function when the second CAR target is expressed by tumor cells. This approach may improve the safety of the therapy in clinical treatment. We tested this concept in a preclinical CAR study targeting ovarian cancer using TAG-72- truncated CD47 dual-targeting CAR-T cells (TAG-72 + ΔCD47 CAR-T) (70). Because CD47 is well-reported as having high expression on ovarian cancer cells but also on many healthy tissues, we designed a ΔCD47 CAR devoid of intracellular signaling domains to predispose cytotoxic impact on normal cells. CD47 CAR helped CAR-T cells engage with CD47^+^ cells, thereby increasing the possibility of TAG-72 CAR to bind tumor cells with even lower TAG-72-expressing levels. This showed that TAG-72 + ΔCD47 CAR-T cells delayed tumor growth in mouse models, compared to single antigen-targeting TAG-72.4-1BB CAR-T cells (70). This “HELP” gate strategy may not only overcome the downregulation of the tumor antigen but may also enhance the clinical safety of dual CAR-T cells.

## “OR” logic gates

### CD30-targeted CARs

CD30 is a member of the tumor necrosis factor receptor family, with expression confined to some subsets of activated T and B lymphocytes (111). CD30 acts as a costimulatory signal to control cell survival and effector functions, while its overexpression has been linked to lymphoma transformation (112, 113). In CTCL skin biopsies, CD30 overexpression is well-documented in primary cutaneous anaplastic large-cell lymphoma, whereas its distribution is highly variable in MF and SS (114, 115). Anti-CD30 drugs, such as brentuximab vedotin, have been evaluated in treating advanced stages of CTCL with a significant clinical response observed (NCT01396070, NCT01578499) (114, 116, 117). In Phase II studies, the overall response rate was over 70% in patients with MF and SS after receiving brentuximab vedotin. However, common adverse effects were neuropathic toxicity observed in 67% of patients (8), which appears to be slowly reversed after dose discontinuation (41.5 weeks on average) (10). The underlying mechanism of peripheral neuropathy is not entirely clear, but is attributed to drug conjugation - monomethyl auristatin E, which disrupts axonal transport between nerve cells (118). Of note, patients experienced disease relapse within 7 months of drug cessation with lower expression of CD30 whereas toxicity symptoms still existed after treatment (10, 119), raising concerns about the long-term efficacy of the CD30 antibody-drug conjugate.

Owing to the high expression on T cell lymphoma, CD30 could be an appropriate CAR target since the toxicity of CD30 mAb therapy was attributable to the conjugated drug. Furthermore, in pre-clinical studies Hombach et al. have showed that CD30 CAR-T cells effectively lyse the MyLa CTCL cell line and suppress tumor growth in CTCL xenograft models (120). CD30 CAR-T therapy has yielded encouraging results with a good safety profile in patients with Hodgkin lymphoma, where patients were relapsed/refractory to prior lines of therapy, including brentuximab vedotin (119, 121, 122). CAR-T fratricide might be mitigated because CD30 expression is normally limited to infrequent T cell subsets upon activation (123). Adverse side effects due to CD30 CAR-T cell destruction of normal healthy cells could potentially still be important, although how extensive this is clinically examined, is uncertain. In this regard, Ramos et al. treated 9 patients with CD30^+^ lymphoma without lymphodepleting chemotherapy prior to CD30 CAR-T infusion. The adverse effects were well tolerated in all patients with no immune impairment nor increased viral infection following CAR-T infusion (121). This may be attributable to the fact that normal T cells have lower levels of CD30 expression than tumor cells and may be insufficient to evoke CAR-T cell killing (120, 124). However, it is unlikely to be just the number of receptors engaged, but also the affinity of the antigen-binding domain and combined signaling strength of the individual components of the whole CAR construct (55, 57, 125). Another aspect that needs to be considered is that CD30 is also expressed by hematopoietic stem and progenitor cells, raising concerns for lasting blood cell aplasia in patients who receive CD30 CAR-T cell treatment (120). Studies in humanized mice showed that hematopoietic stem and progenitor cells retain their differentiation capabilities and resist CD30 CAR-T cell attack, confirming tolerated on-target/off-tumor toxicity on the primary bone marrow cell population (120).

In an attempt to achieve sustainable responses with manageable side effects, many ongoing clinical trials have been registered to evaluate CD30 CAR-T efficacy in treating Hodgkin and non-Hodgkin lymphoma (NCT02917083, NCT01316146, NCT03049449, NCT02690545), but none are studying CTCL treatment-specific diseases.

In CTCL, only certain lineages of malignant T cells express CD30 at variable levels (114). Thus, targeting CD30 alone may not cover the entire tumor population. Also, relapsed MF patients generally express CD30 at lower levels after anti-CD30 targeted drug treatment, suggesting either antigen loss or heterogeneity of cell surface expression may be preventing the complete eradication of these potentially tumorigenic cells (10). Further work is required to assess CD30 expression levels on circulating malignant T cells but importantly also normal T cells to assure the safety and efficacy of the therapy.

### CCR4-targeted CARs

The chemokine receptor CCR4 is expressed in various T cell subpopulations and is upregulated in many T cell malignancies, including CTCL (126, 127). In MF and SS patients, CCR4 is strongly expressed by malignant T cells in both skin and peripheral blood compartments of the disease (22, 128). CCR4 may facilitate the migration of malignant T cells through the vasculature to the skin lesions (16). Indeed, CCR4 ligand, C-C motif chemokine ligand 17, is significantly increased in MF/SS patients compared with patients suffering from benign inflammatory dermatoses (129). As such, mogamulizumab - the first Food and Drug Administration-approved CCR4-targeted drug - has been used to treat relapsed and refractory CTCL (128, 130, 131). CCR4 CAR-T cells were first evaluated by Perera et al. who showed anti-tumor potency against CTCL CCR4^+^ cell lines (HH and HuT78) *in vitro* (132). In xenograft murine models, CCR4 CAR-T therapy effectively eradicated cell leukemia and mice remained disease-free at the end of the study (however this was only 12 weeks duration), while the tumor continued growth in all mice treated with control T cells (132).

Notably, CCR4 is also found on regulatory T cells (Tregs) – a major player that inhibits immune activity in the tumor microenvironment by producing immunosuppressive cytokines, such as IL-10 and transforming growth factor-β2 (133). Tregs also constitute a physical barrier that hampers the penetration of immune cells into the tumor microenvironment, which in turn, inhibits the immune response against tumors (3). Accordingly, increasing numbers of Tregs were associated with unfavorable survival outcomes in several tumors (3). In CTCL patients, over 90% of circulating Tregs are CCR4^+^. Accordingly, CTCL patients treated with mogamulizumab observed a decreased number of CCR4^+^ Tregs that may be instrumental for boosting immunity *via* restoring NK cell and CD8 T cell function (128). In line with this study, Kim et al. also showed Treg ablation may rescue CD8 cytotoxic T cell activity and increase NK cell number in the lung tumor environment, resulting in enhanced anti-tumor immunity (134). Using KM2760, the CCR4 mAb to treat MF/SS patients, Ito et al. showed a reduction in CCR4-expressing Treg number in CTCL mouse models, which again improved host immune response to the tumor cells (135).

Hence, apart from targeting tumor cells, CCR4 CAR-T cells may also eliminate Tregs to facilitate the migration of immune cells into the tumor microenvironment, thereby augmenting anti-lymphoma activity of the therapy. Given that CCR4 is distributed mainly in specific CD4^+^ T cell subsets (Th2, Th17) (136, 137), engineering CCR4 CAR-T cells from the CD8^+^ T cell population may avoid product contamination with malignant T cells and mitigate fratricide between therapeutic cells under the “OR” logic gate scenario. This strategy allows CAR-T cells to trigger cytotoxic responses upon engagement with either antigen 1 (CCR4) or a second antigen. In terms of safety, adverse events are manageable following mogamulizumab administration (130, 138). CCR4 upregulation has been reported in activated circulating memory T cells and skin epidermis in response to an inflammatory stimulus (129). As CAR-T cells are more sensitive at recognizing cells with lower CCR4 expression than mAbs (139), to ascertain safety in patients, an intensive evaluation of CCR4 expression in healthy people (both skin and peripheral blood) is essential prior to clinical implementation. The second CAR target needs to be highly specific for the tumor to alleviate toxicity of non-tumor cells.

### TAG-72-targeted CARs

Tumor-associated glycoprotein 72 (TAG-72), is a high molecular weight transmembrane glycoprotein-like mucin, that has been described as an oncofetal marker for many types of adenocarcinomas (140–142). As TAG-72 expression is almost totally absent in normal adult tissue except in limited postovulatory endometria (142, 143), it could be an ideal target for adoptive immunotherapy. TAG-72 CAR-T cells have demonstrated efficacy in killing certain types of solid tumor cell lines *in vitro* (70, 143, 144). In xenograft models of ovarian cancer, repeated infusion of TAG-72 CAR-T cells significantly extended overall mice survival compared to single treatment (55 days versus 30 days benefit) (143). It should be noted, however, that reduced expression of TAG-72 was observed on relapsed ovarian cancer cells in mice following TAG-72 CAR treatment (143), therefore engineering a second CAR may mitigate potential antigen escape. Phase I trials (C-9701 and C-9702) of a first-generation TAG-72 CAR-T therapy showed a good safety profile in patients with colorectal cancer, but CAR-T persistence was limited (≤14 weeks) (145). As expected, the trafficking of TAG-72 CAR-T cells to the liver and rectum was observed, however, there were no radiologic tumor responses (145). These patients also produced antibodies to the CAR-T cells because of remnant mouse immunogenicity epitopes in the scFv, causing the trials to be stopped (145). A humanized scFv directed to TAG-72 may overcome this issue, which could increase the safety of TAG-72 CAR-T therapy (70).

Although TAG-72 has drawn broad interest as a therapeutic candidate in the treatment of solid tumors, the characteristics of TAG-72 are not well understood in the treatment of blood-based cancers. A study on this subject found that Jurkat T cells derived from acute T cell leukemia, strongly express TAG-72 levels on their surface (146). Given this finding, investigating the extent of TAG-72 expression in CTCL is warranted.

## “NOT” logic gate

### CD37-targeted CARs

CD37, a member of the transmembrane 4 superfamily, typically acts as a transduction signal to mediate cell death. CD37 is predominantly expressed in mature B cells and at lower levels in T cells, NK cells, and monocytes (147). Interestingly, recent studies have reported CD37 expression in CTCL patients (148, 149), suggesting it’s potential as a therapeutic target. AGS67E, an anti-CD37 antibody-drug conjugate, has demonstrated a partial clinical response to CTCL disease with a favorable safety profile (Phase I trial) (148–150). Scarfo et al. also showed the CTCL patient-derived cell line, HuT78, was killed after being exposed to CD37 CAR-T cells in *in vitro* cytotoxicity assays (148). The concept of a CD37 CAR targeting T cell lymphoma would have minimal CAR-T cell fratricide and T cell aplasia because of the absent or weak expression of CD37 in normal T cells. A phase I trial of CD37 CAR-T cells is currently active for the treatment of patients with hematologic malignancies (NCT04136275).

Despite these advantages, CD37 CAR-T cells may deplete normal mature B cells from the peripheral circulation, leading to humoral immunodeficiency following therapy. Multi-targeted CARs under the “NOT” logic gate system may offer an alternative strategy to avoid this B cell clearance. By design, a second CAR is incorporated for immune inhibitory receptors instead of a costimulatory domain, thereby impeding the first CAR activation (**Figure 8**). Cytotoxic T lymphocyte-associated protein 4 or programmed cell death protein 1 were exploited to constitute an inhibitory CAR (iCAR) (151). Tao et al. designed multipronged CAR-T cells: CD19 CAR to target B cell leukemia; the second CAR was a programmed cell death protein 1-based iCAR specific for HLA-C (151, 152). HLA is predominantly found on normal tissue, but cancer cells often downregulate HLA expression on their surface to escape immune surveillance (153, 154). The group reported that the iCAR released less cytokines and presented lower toxicity on normal B cells. In the xenograft mouse model, iHLA-CD19-CAR-T cells selectively killed “on-target” malignant B cells (CD19^+^, HLA-C^+^), although normal B cells (CD19^+^, HLA-C^-^) remained detectable. This result was explained by the negative signaling delivered by the inhibitory programmed cell death protein 1 incorporated with the HLA-C iCAR domain, which suppressed CAR-T cell activation quickly following encounter with normal B cells. To increase the safety and efficacy of these cells, the iCAR target should be highly expressed on normal cells, but expressed only at restricted levels on tumor cells. Given that most previous studies assess CD37 expression *via* immunostaining in tissue samples, further investigations should examine CD37 levels in peripheral blood in CTCL patients as CAR-T infusion would easily access the circulating malignant T cell population first.

## Conclusion

CTCL disease remains a significant challenge with low survival in patients with advanced disease. CAR-T cells recently revealed remarkable clinical success in the treatment of B cell malignancies and thus present as a potential treatment option for T cell malignancies, particularly CTCL. Although several studies have reviewed a broad aspect in potential strategies for treating T cell lymphoma, none were specific to CTCL disease. The translation of CAR-T cells in the treatment of CTCL faces significant challenges due to tumor heterogeneity and the phenotypic similarities between normal and T cell lymphoma (risk of T cell aplasia, CAR-T fratricide, and product contamination). Selecting appropriate CAR targets will play a fundamental role in the efficacy and safety of potential therapies. Here, we reviewed six potential antigens targeted by CAR-T cells in CTCL, namely CD4, CD47, CD30, CCR4, TAG-72, and CD37. Single-antigen targeting is unlikely to cover the entire tumor population (due to tumor heterogeneity) in patients. Multi-specific receptors in CAR-T cells, derived from the CD8^+^ subset, would likely be necessary to offset the disease’s heterogeneity and avoid contamination of the therapeutic product with leukemic CD4^+^ T cells. Different logic gate formats can be tailored to strike the balance between the safety and efficacy of the therapy. To offset tumor heterogeneity and enhance anti-tumor effector efficacy, the “OR” logic gate activating CAR-T cells in the presence of an individual cognate antigen, could be employed. Developing dual CARs under the “HELP” logic gate may improve tumor recognition but still ensure the safety of the CAR therapy. “NOT” logic gate is an alternative configuration of the “HELP” logic gate that may enable CAR-T cells to distinguish between T cell lymphoma and normal cells, thus avoiding cytotoxicity on normal cells. The next frontier of future research will be reassessing the expression of target antigens in both CTCL disease compartments (skin and peripheral blood) and optimizing CAR constructs of potential antigen combinations to develop a novel CAR-T that is curative for this aggressive disease.

## Author contributions

All authors listed have made a substantial, direct and intellectual contribution to the work, and approved it for publication.

## Acknowledgments

VT is in receipt of a VinGroup Science and Technology Scholarship and acknowledges the financial support from the VinUniversity, VinGroup Scholarship Program, Vietnam. VT also acknowledges the kind help and support of everyone in the Cartherics team. The figures were created with Biorender.com.

## Conflict of interest

All authors, except V.T, are paid employees or advisors of Cartherics Pty Ltd, and hold equity in the company.

The remaining author declares that the research was conducted in the absence of any commercial or financial relationships that could be constructed as a potential conflict of interest.

## Publisher’s note

All claims expressed in this article are solely those of the authors and do not necessarily represent those of their affiliated organizations, or those of the publisher, the editors and the reviewers. Any product that may be evaluated in this article, or claim that may be made by its manufacturer, is not guaranteed or endorsed by the publisher.
